# Addressing HIV and Substance Use Health Disparities among Racial/Ethnic Minority Individuals

**DOI:** 10.1007/s11904-025-00738-z

**Published:** 2025-04-04

**Authors:** Jamie V. Saunt, Kate M. Kelley, Corrilynn O. Hileman, David L. Hussey, Ann K. Avery

**Affiliations:** 1 Mandel School of Applied Social Sciences, Case Western Reserve University, Cleveland, OH USA; 2 Department of Psychiatry, Center for Addiction Medicine, MetroHealth Medical Center, Cleveland, OH USA; 3 Department of Medicine, Division of Infectious Diseases, MetroHealth Medical Center, Cleveland, OH USA; 4 School of Medicine, Case Western Reserve University, 2500 MetroHealth Drive, Cleveland, 44109 USA

**Keywords:** HIV, Substance use disorder, Racial minority, Ethnic minority, Harm reduction

## Abstract

**Purpose of Review:**

Advances in HIV testing, prevention, and treatment, alongside increased awareness and harm reduction efforts for substance use disorder (SUD) have improved care and treatment access over the past decade. However, racial and ethnic minorities with SUD and HIV or at risk for HIV still face disproportionately high health disparities. Understanding and addressing the reasons behind these disparities is crucial.

**Recent Findings:**

Structural and systemic barriers continue to negatively impact minoritized communities, due to lack of access to care, mistrust, and feelings of ostracization. Disconnected systems for HIV and SUD treatment complicate combined care. Delays in HIV diagnosis and viral suppression reduce life expectancy for minority populations by around 10 years.

**Summary:**

Healthcare systems need to become more integrated, accessible, and culturally welcoming to marginalized communities. Promising interventions utilizing technology, harm reduction, and mobile service delivery can reduce barriers and improve outcomes for minority individuals.

## Introduction

The intersection of HIV and substance use presents multiple challenges in public health, especially among racial and ethnic minority populations. According to 2022 data published by the Substance Abuse and Mental Health Services Administration (SAMHSA), the percentage of people aged 12 or older who use illicit drugs is highest among multiracial people, Black people, and American Indian or Alaska Native people. Yet, treatment utilization is highest among White people, demonstrating the gap in access to services and treatment utilization for substance use disorder (SUD) for minority populations [1]. Minority populations are also disproportionately impacted by the HIV epidemic. According to existing research, HIV testing and treatment is lower for Black and Latinx populations, while negative health outcomes from HIV are higher [2–5]. When both SUD and HIV co-occur in racial and ethnic minority groups, health disparities become more apparent. Even with advancements in HIV testing, prevention, and treatment, these disparities persist, exacerbated by complex socio-economic, cultural, and systemic factors.

Addressing the racial and ethnic disparities among people with HIV and SUD requires a comprehensive approach that encompasses improving access to care and addressing systemic barriers. As research continues to identify barriers that contribute to these health disparities, it is essential to develop and implement strategies that put the burden of care on the system, rather than on the individual. Literature identifies what the barriers are and why these disparities persist; researchers, healthcare providers, and policy makers are tasked with the need to adjust our systems of care to adequately meet the needs of these marginalized populations. This paper serves as an overview of the current literature, examining the prevalence of HIV and SUD in minority populations, services available, disparities in outcomes, and the continued barriers individuals from racial and ethnic minority groups face around being diagnosed with HIV and obtaining treatment for HIV and SUD. See Table 1 for an overview of dimensions through which racial and ethnic disparities manifest in the context of HIV, Substance Use Disorder, and related health issues.

### Prevalence

According to the Centers for Disease Control, in 2022, Black and Latinx individuals made up 12% and 18% of the US population but accounted for 37% and 33% of new HIV infections, respectively. Thus, Black and Brown people accounted for 70% of new HIV infections, while White people made up 61% of the population, but accounted for only 24% of new HIV infections [6]. Structural racism is fundamental to understanding prevalence. Regan et al. found that Black people with HIV (PWH) reported more drug use and more drug-related negative consequences when compared to White PWH [7]. Despite the increase in HIV prevention efforts, these disparities continue to persist, with young, minority men who have sex with men (MSM) facing disproportionately high rates of HIV infection [8, 9]. In addition, while minority communities use less injection drugs, they face higher rates of overdose deaths, especially in counties of high-income inequality [10]. Older age compounds disparities in minority communities. In 2020, the overdose death rate among Black males older than 65 was almost seven times higher than non-Hispanic White males [10]. Thus, prevalence can be examined through the lens of racial disparities, and the current literature highlights that multiple factors lead to racial disparities.

More specifically, Prosperi et al. examined HIV infection in Florida - a state that is the third most populus in the United States but has the highest rate of HIV infection - by utilizing the Florida Department of Health’s Syndromic Tracking and Reporting System (STARS) database and causal artificial intelligence. Their research found that education, income, violent crime, smoking, alcohol use, and living in a rural area were all associated with risk of HIV infection [3]. Similarly, while people who inject drugs are at increased risk of HIV overall, Jin et al.’s examination of data from the National HIV Surveillance System (NHSS) and the US Census Bureau’s American Community Survey cited education, poverty, and low income as particular risk factors among PWID [11].

In general, racial disparities in HIV have not been attributed to higher levels of sexual risk behavior, but rather higher prevalence of HIV infection among sexual partners [12]. However, substance use, especially stimulant use (primarily cocaine and methamphetamine), are associated with higher rates of sexual risk for HIV. To date, rates of stimulant use are lower in black populations compared to White and Latinx. However, when used, its negative impact was greater, partially due to the prevalence of sexualized stimulant use in MSM “sex parties” [13, 14] and was associated with higher levels of transactional sex, diagnosed STIs and lack of use of HIV pre-exposure prophylaxis for HIV (PrEP) [15].

Prevalence is also setting-specific to areas that have racially driven higher rates of minorities, such as jails and prisons. Brewer et al. completed a systematic review assessing the impact of HIV and associated factors among criminal justice-involved Black sexual and gender minority populations, finding that the dual epidemics of HIV and incarceration for the Black community has significant impact. Criminal justice involvement is associated with a greater likelihood of STI’s among Black MSM, and although criminal justice settings offer HIV testing, these settings are not conducive to continuous HIV care [16]. Finally, while low income is cited as a factor, no cost care is not necessarily the sole solution: within the veteran community utilizing free care through the VA, the highest HIV prevalence was found to be among Black and Latinx veterans with a Hepatitis C virus diagnosis, between the ages of 50 and 64 [17]. Furthermore, even among groups where healthcare utilization is high, there continue to be high rates of HIV and disability, potentially due to stigma, medical mistrust, and lower levels of patient satisfaction [18].

These studies show that prevalence is compounded by intersecting identities and status. The combination of factors is more impactful than any one individual issue, with some holding greater weight than others. Thus, a solution to decreasing prevalence requires a coordinated, systemic approach to make progress towards disparity reduction.

### Services

Because of the increase in health disparities among racial and ethnic minority groups and people who use substances, it is important to examine the settings in which people do and do not receive services and support for HIV testing and care and SUD treatment. Des Jarlais et al. emphasized four pillars of HIV work: counseling and testing, risk reduction services, access to antiretroviral therapy (ART), and drug abuse treatment [19]. Lack of access to services is a major barrier to receiving care.

A foundational challenge in providing HIV services (including screening, prevention, and treatment) is lack of integration of these services in SUD treatment programs, even though injection drug use remains a risk factor for HIV transmission and acquisition. Utilizing data from the National Survey of Substance Abuse Treatment Services, Ware et al. evaluated 10,415 residential SUD treatment facilities and found not only regional differences among coordinated HIV and substance use services (Midwest and West less likely to provide these services together), but also organizational differences, such as payment and accreditation [20]. As there is no national gold standard for screening, prevention, and treatment, residential treatment facilities varied widely in access to care, with those who accepted Medicaid and held accreditation offering more comprehensive services. Furthermore, private for-profit and government facilities were more likely to offer HIV services than private nonprofit organizations, a finding that requires more research, but may be related to reimbursement rates and payment models [20]. A comparison among racial and ethnic groups from the State Unintentional Drug Overdose Reporting System (SUDORS) in 2020 found that previous substance use treatment was lowest for Black persons (8.3%) [10]. West et al. examined SUD treatment among PWH using data from the National Survey on Drug Use and Health (NSDUH) and found that more than three-quarters reported no past-year treatment of SUD [21].

For people who inject drugs and are not engaged in residential treatment, a fundamental tool for HIV prevention and lowering transmission is syringe exchange. According to a study by Rosen et al., new HIV diagnoses attributed to injection drug use decreased by 53.9% from 2008 to 2020, partly due to extensive harm reduction efforts such as syringe exchange programs [22]. Unfortunately, Salow et al. found in their survey of syringe exchange programs in King County, WA that many people who inject drugs from racial and ethnic minorities were underrepresented in utilization of the programs [23]. Poiter et al.’s 2014 review showed how integrating supervised injection sites– a step beyond syringe exchange programs - may connect those who are often most marginalized within the SUD community with important services [24].

Creative approaches have been proposed to increase access to and receipt of SUD and HIV services among racial and ethnic minority groups. These interventions, including multi-faceted strategies to improve access to services like outreach using technology and social media, focus on engagement through creating welcoming environments, and use of peer support, have had promising outcomes [8, 9, 25, 26]. Youth at risk might benefit from technology-forward programs based in a holistic and enjoyable or fun model of care constructed in culturally consistent frameworks [8]. Importantly, as Gwadz et al. noted in their 2022 qualitative study interviewing 46 individuals with non-suppressed HIV viral load, programs should highlight the systemic and structural factors that drive health behavior in order to foster engagement and behavior change (Intervention Innovations Team integrated conceptual model) [27]. Reforming and creating integrated programs of HIV and substance use care with a backbone of welcoming, culturally aware, and responsive staff who prioritize meeting basic needs can help to address the disparities in access to HIV and SUD care.

### Outcomes

Improving outcomes in PWH with substance use disorder from minority communities starts with expanding HIV testing opportunities. Unfortunately, HIV testing rates are lower among racial and ethnic minorities. Among people who use drugs admitted to a large medical center, Black and Latinx patients were less likely to receive HIV testing during admission than White patients [adjusted OR 0.69, 95% confidence interval (0.59–0.83) and 0.68, (0.55–0.84), respectively], even though rates of testing were low overall (10%) [5].

A salient outcome once an individual is diagnosed with HIV is suppression of the virus on antiretroviral treatment (ART). To achieve durable virologic suppression, there is likely an intersectionality of factors that contribute to success including housing status, income level, medical insurance status, substance use and mental health status [28]. Further, the factors that matter most, and even the direction of the effects, depend on race, ethnicity and gender [28]. Evaluating risk in this context makes sense as PWH often hold multiple marginalized identities.

Several specific factors are associated with lack of viral suppression among PWH who use drugs and these are more prevalent in individuals from racial and ethnic minority communities. Use of specific drug classes such as methamphetamines increases risk of uncontrolled HIV viremia. Among transwomen with HIV living in San Francisco, the majority of who were transwomen of color, methamphetamine use was associated with a detectable or unknown HIV viral load [29]. On the other hand, there is some evidence that high intensity cannabis use increases the likelihood of virologic suppression in PWH who use cocaine or injection drugs [30, 31]. Delay in ART initiation in some minority communities may also contribute to delay in virologic suppression. While early ART initiation has increased substantially over the years since the HIV treatment guidelines were updated to recommend ART for all PWH regardless of CD4 count, being a non-Hispanic Black person, residing in the South census region, being a male with injection drug use HIV acquisition risk, and history of SUD were all associated with delayed ART as well as a delay in virologic suppression in the North American AIDS Cohort Collaboration on Research and Design (NA-ACCORD) clinical cohorts in the US [32]. Compounding this risk is that there is often a substantial gap in substance use treatment engagement. In women with and without HIV in Southern states who use drugs, only 25% reported substance use treatment. While retention in HIV care did not differ by substance use treatment, HIV viral suppression was significantly higher in women who reported substance use treatment at enrollment [33].

Linkage to HIV care and ART adherence go hand-in-hand with HIV virologic suppression. Among majority Black (73%) PWH with substance use disorder, recent drug use, high levels of medical mistrust, and not completing high school were associated with lower likelihood of early linkage to care after hospitalization [34]. Early linkage to care is important as this was associated with ongoing engagement in care at 6 months post-hospitalization [34]. In young PWH, ART adherence was negatively associated with depression, trauma, adverse childhood experiences, cannabis, and stimulant use, suggesting that interventions that provide ongoing mental health and substance use treatment are important for engagement in HIV treatment [35]. Use of technology such as videoconferencing was identified as a favorable way to increase capacity and accessibility to these services among PWH aged 18–29, but interventions should account for varying needs at different stages of life [35, 36].

There are clear differences in HIV outcomes by race and ethnicity in PWH who use drugs. Furthermore, while PWH receiving ART are living longer than ever before, there are disparities in survival in some key populations including among Black MSM compared to White MSM and people with and without a history of injection drug use; survival differences of approximately 10 years exist between these groups [37].

### Barriers and Future Directions

In identifying barriers and future directions, it is essential to look holistically at the United States and the structural inequities that exist which continue to propagate disparities within the medical and larger social systems. In healthcare alone, the predominance of the fee-for-service model and the splintering of health care resources, in addition to differences between States in terms of insurance coverage and access to clinicians, makes the already vulnerable population of PWH who use substances more at risk of inferior care provision. While access to health care is often cited in the literature as an issue for people who use substances and are at risk of HIV or are PWH, the underpinning of access is much more nuanced than simply increasing the number of care providers. Factors contributing to disparities for young, minority MSM specifically include poverty, systemic racism, food insecurity, housing instability, limited health insurance, and low health literacy [8, 9]. Black, Latinx, and indigenous people are diagnosed with HIV later than their White counterparts, due to healthcare barriers such as lack of insurance, lack of transportation, and inability to take time off work to visit traditional business-hour healthcare services [38]. Loza et al. examined health disparities in El Paso County, finding that among Latinx MSM, half of participants had no health insurance and one third of participants paid for healthcare out of pocket [39].

Barriers for ethnic and racial minority groups are not limited to HIV and SUD care. Minoritized populations have a greater prevalence of chronic conditions, such as hypertension and diabetes, than their White peers [40]. The COVID-19 pandemic also illuminated ongoing disparities among healthcare access, services, and outcomes [41]. It is tempting to place all the blame on cost and insurance coverage; however, the BESURE study, which examined the unequal prevalence of HIV, hepatitis C, and mental health conditions in persons who inject drugs, found that insurance status was not a concrete explanation of disengagement in care [18]. Specifically, individuals with low healthcare utilization rates were those employed or uninsured, whereas those insured and unemployed had higher utilization of care [18]. These findings highlight that the structure in which one receives care (e.g. insurance model, limited clinic hours) was as important a consideration as healthcare coverage. Lack of health care utilization among older individuals was also an important finding of the BESURE study, which reflected the historic distrust Black individuals may feel towards the health care system in general [18]. Simply having insurance and access to care does not necessarily translate into utilization for individuals from racial and ethnic minority groups. Minority MSM report feeling ostracized by their racial communities due to homophobia and alienated by their LGBTQ + communities due to racism [8]. Barriers to testing, care, and treatment continue to negatively impact Black and Latinx MSM due to healthcare systems that are challenging to navigate and unwelcoming, compounded by transportation struggles and prioritizing meeting basic needs over healthcare appointments [2, 20, 24].

Policies that reinforce health inequities continue to be politically popular [42]. The “War on Drugs” has led to mass incarceration of Black and Brown communities, disrupting social networks and communities and contributing to increased HIV risk [16]. The disproportionate impact of incarceration on communities of color is deeply intertwined with healthcare challenges regarding HIV. Incarceration is associated with lack of trust in healthcare, lack of engagement with physicians, and higher feelings of stigmatization, leading to higher risk of undiagnosed HIV infection [43].

Pinto et al.’s review of integrated HIV and SUD services emphasized that silos of care lead to increased barriers to access, including the non-medical issues that increase stress and chaos in the already difficult lives of PWH who use substances. Barriers should not be labeled as individual issues, but rather as structural inequalities [44]. PWH who use substances have complicated and often challenging lives due to both societal inequities out of their control and individual decisions made in stressful environments. Ramirez-Ortiz et al. describes the lack of utilization of HIV services in Miami-Dade County as “competing life priorities”– in this study, female patients deprioritize their own health needs as other needs (childcare, food, transportation) are seen as more tangible and prominent [45]. Patients often make the best choice at a moment in time given their situation. One example of healthcare structures actually exploiting poverty and vulnerability was shown in a study by Filippone et al. in which patients were paid in cash by pharmacies in exchange for leaving their ART prescriptions unfilled [26]. Systemic and policy barriers such as inadequate healthcare infrastructure and disconnected systems for HIV and SUD treatment further compound challenges for marginalized populations [27, 46].

Perhaps more simply, for many ethnic and racial minorities, a barrier to accessing services is a basic human emotion: fear. In a qualitative study detailing the experience of Black women who use cocaine in the greater Nashville, TN area, fear was a reoccurring theme. Fear stemmed from the possibility of losing custody of their children, losing entitlements, change, not understanding what the treatment experience will consist of, and being assumed to have been diagnosed with HIV [47]. With this fear, a sense of powerlessness was also prominent. As stated earlier in this review, the current design of healthcare places the burden of care on the patient, and not on the system itself. This is an increasingly heavy weight on PWH who use substances, and one that is not simply solved by expanding services or increasing funding.

## Conclusion

It is tempting to point fingers when reviewing the challenges faced by racial and ethnic minority groups who experience substance use and HIV risk or treatment. We believe that the current literature presents opportunity, rather than blame, and research detailed in this review provides a path forward. To start, refortifying our foundation of holistic person-centered health care would help address some barriers at the root of disparities. Furthermore, clinicians and policymakers’ recognition of the role systemic and structural racism and discrimination play in HIV and substance use care for minority populations can open a discussion of innovative program delivery systems. Training more members within marginalized communities to participate in education and direct care, in addition to harnessing the role of technology, mobile service delivery, and primary care integration are promising steps. Honesty about the barriers we face in this work can help propel us to not only avoid repeating mistakes, but also make significant strides to best care for patients in our community.

**Table 1:** Conceptual framework of ethnic and Racial disparities in HIV, substance use disorder and related factors

Factors	Description	Impact on Racial/Ethnic Minorities
HIV Risk of Transmission	The likelihood of transmitting HIV to others.	Higher in minority communities due to lower rates of viral suppression
Viral Suppression	The ability to reduce viral load to undetectable levels with ART.	Lower rates of viral suppression among minority groups.
Risk of Infection	The likelihood of acquiring HIV.	Increased due to higher prevalence in communities and lower prevention resources.
HIV Testing	Access to and utilization of HIV testing services.	Lower testing rates and late diagnoses in minority populations.
Substance Use Disorder	Substance use and method of substance use, impacting HIV risk factors and health outcomes.	Higher prevalence of substance use disorders among racial/ethnic minorities.
Stigma	Negative social attitudes towards people with HIV and people who use substances.	More pronounced stigma in minority communities, leading to poorer health outcomes.
Access to Treatment	Availability and utilization of HIV and substance use treatment services.	Barriers such as insurance coverage, transportation, and provider bias.
Barriers to Treatment	Challenges faced in obtaining necessary medical care.	Includes financial, structural, and cultural barriers.
Quality of Care	Decreased service engagement & retention. Siloed, fragmented, and inferior service provision.	Higher levels of client mistrust; inadequate behavioral health and harm reduction service provision; poorer patient outcomes.
Healthcare Policies	Legislation and policies affecting HIV and substance use prevention, care, and treatment.	Disparities in policy implementation and effectiveness.
Culturally Connected Care	Healthcare that is culturally sensitive and relevant to the patient’s background.	Lack of culturally competent care exacerbates disparities.

This table is designed to provide a clear and comprehensive overview of the various dimensions through which racial and ethnic disparities manifest in the context of HIV, Substance Use Disorder and related health issues

## Key References

- Freese TE, Padwa H, Oeser BT, Rutkowski BA, Schulte MT. Real-World Strategies to Engage and Retain Racial-Ethnic Minority Young Men Who Have Sex with Men in HIV Prevention Services. AIDS Patient Care STDs. 2017;31(6):275–281. doi:10.1089/apc.2016.0310
-
  - Summit bringing together multiple providers from innovative programs across the United States to assess practices most effective at engaging and retaining young MSM. Highlighted four strategies: utilizing peers and creating welcoming environments, utilizing technology such as social media and chat groups, incorporating fun through using food and community spaces, and providing holistic care through addressing multiple life challenges for higher treatment engagement.
- Brewer R, Ramani SL, Khanna A, et al. A Systematic Review up to 2018 of HIV and. Associated Factors Among Criminal Justice-Involved (CJI) Black Sexual and Gender Minority Populations in the United States (US). J Racial Ethn Health Disparities. 2022;9(4):1357–1402. doi:10.1007/s40615-021-01076-7
-
  - Systematic review addressing Black MSM and Black Transgender women with HIV and criminal justice involvement. Highlighted criminal justice setting as important arena for testing and diagnosis, but poor follow through for continuous care.
- Filippone P, Serrano S, Campos S, et al. Understanding why racial/ethnic inequities along the HIV care continuum persist in the United States: a qualitative exploration of systemic barriers from the perspectives of African American/Black and Latino persons living with HIV. Int J Equity Health. 2023;22(1):1–20. doi:10.1186/s12939-023-01992-6
-
  - Qualitative analysis of Black and Latinx PWH with unsuppressed viral load indicating impact of chronic poverty, housing instability, experiences with providers, pharmacies, and healthcare settings on engagement in HIV care and ART adherence.
- Rivera AS, Rusie LK, Feinstein MJ, Siddique J, Lloyd-Jones DM, Beach LB. Intersectionality-informed analysis of durable viral suppression disparities in people with HIV. AIDS. 2023;37(8):1285–1296. doi:10.1097/QAD.0000000000003565
-
  - Retrospective cohort analysis of electronic health records informed by intersectionality showing that social factors, sexual orientation, gender identity, race and ethnicity interact to result in different likelihoods of durable virologic suppression of HIV.
- Li J, Humes E, Lee JS, et al. Toward Ending the HIV Epidemic: Temporal Trends and Disparities in Early ART Initiation and Early Viral Suppression Among People Newly Entering HIV Care in the United States, 2012–2018. Open Forum Infect Dis. 2022;9(8):ofac336. Published 2022 Jul 22. doi:10.1093/ofid/ofac336
-
  - Retrospective cohort study utilizing data from North American AIDS Cohort Collaboration on Research and Design (NAACORD) from 2012 to 2018, showing factors associated with delayed ART and virologic suppression.
- Althoff KN, Chandran A, Zhang J, et al. Life-Expectancy Disparities Among Adults With HIV in the United States and Canada: The Impact of a Reduction in Drug- and Alcohol-Related Deaths Using the Lives Saved Simulation Model. Am J Epidemiol. 2019;188(12):2097–2109. doi:10.1093/aje/kwz232
-
  - Modeling study showing life expectancy disparity in people with HIV in the US and Canada based on key factors including race, sex, history of injection drug use.

## Data Availability

No datasets were generated or analysed during the current study.
